# ﻿Three novel species and a new record of Pleosporales (Didymellaceae, Roussoellaceae) from China

**DOI:** 10.3897/mycokeys.113.139934

**Published:** 2025-02-12

**Authors:** Shi-Qi Guo, Chada Norphanphoun, Kevin D. Hyde, Sha-Min Fu, Jing-E Sun, Xing-Chang Wang, Jiao-Jiao Wu, Fatimah Al-Otibi, Yong Wang

**Affiliations:** 1 Department of Plant Pathology, Agricultural College, Guizhou University, Guiyang, 550025, China Guizhou University Guiyang China; 2 Department of Botany and Microbiology, College of Science, King Saud University, P.O. Box 22452, Riyadh 11495, Saudi Arabia King Saud University Riyadh Saudi Arabia; 3 Centre of Excellence in Fungal Research, Mae Fah Luang University, Chiang Rai 57100, Thailand Mae Fah Luang University Chiang Rai Thailand

**Keywords:** 3 new taxa, Ascomycota, diversity, morphology, *Neoroussoella*, *Roussoella*, *Xenodidymella*

## Abstract

We are investigating saprobic Ascomycota in Guizhou Province, China. Fungal specimens collected from dead wood were identified based on morphological characteristics and multi-gene phylogenetic analyses of ITS, SSU, LSU, *β-tubulin*, *ef1-α*, and *rpb2* sequence data. Three novel species, *Neoroussoellaguizhouensis*, *Roussoellaguizhouensis*, and *Xenodidymellaguizhouensis*, are introduced, along with one new geographical record, *Xenoroussoellatriseptata*. This study contributes to our understanding of the diversity of Ascomycota in Guizhou Province and the classification in Roussoellaceae.

## ﻿Introduction

Pleosporales, the largest and most diverse order within the class Dothideomycetes, encompasses over 20,000 species across more than 220 families (Hyde et al. 2024a; Pem et al. 2024). Members of this order play key ecological and economic roles, including functioning as plant pathogens, saprobes, and endophytes on various host plants (Ariyawansa et al. 2020). Recent surveys indicated that Guizhou province is a hotspot for fungal biodiversity, particularly within its forested and bamboo-dominated ecosystems (Wijayawardene et al. 2021a; Sun et al. 2023; Yang et al. 2023; Zhang et al. 2023). With its subtropical climate, diverse topography, and high humidity, this region offers a fertile environment for fungal growth. Our study focuses on fungal specimens collected from decaying wood and bamboo culms in Guizhou province, China.

Roussoellaceae was defined with accommodating *Neoroussoella*, *Roussoella*, and *Roussoellopsis* (Liu et al. 2014). The family has been expanded to include more genera such as *Appendispora*, *Cytoplea*, *Elongatopedicellata*, *Immotthia*, and *Pararoussoella* (Bizzozero et al. 1885; Barr et al. 1987; Hyde et al. 1994; Ariyawansa et al. 2015; Jaklitsch et al. 2016; Wanasinghe et al. 2018; Pem et al. 2024). Although Jaklitsch et al. (2016) suggested that Roussoellaceae should be synonymous with Thyridariaceae, this conclusion has not been universally accepted, and other studies supported Roussoellaceae as a distinct family (Liu et al. 2015; Tibpromma et al. 2017; Wijayawardene et al. 2017; 2018; Hyde et al. 2018). Currently, 13 genera are recognized within Roussoellaceae, based on comprehensive morphological and molecular data (Hongsanan 2020; Wijayawardene et al. 2021b; Li et al. 2023; Hyde et al. 2024a).

Didymellaceae is the largest and most diverse group in Pleosporales (Hyde et al. 2024b). This family comprises three main genera: *Ascochyta*, *Didymella*, and *Phoma*, and several allied phoma-like genera (Gruyter et al. 2009). Didymellaceae species are widely distributed across various hosts and habitats, with many species being important plant pathogens (Chen et al. 2015). Some species have quarantine significance, posing substantial challenges to regulatory organizations tasked with managing plant health and preventing the spread of plant diseases. Additionally, several species within Didymellaceae exhibit saprobic and endophytic lifestyles, colonizing a wide range of hosts (Aveskamp et al. 2008; Pem et al. 2024).

The present study introduced Roussoellaceae and Didymellaceae taxa isolated from dead wood in Guizhou province, supported by their distinct morphological and phylogenetic characteristics. We use a polyphasic approach as guidelines for introducing new species (Maharachchikumbura et al. 2021), and this comprehensive analysis enhances our understanding of fungal diversity in the region and contributes valuable insights into their taxonomy and ecological roles.

## ﻿Materials and methods

### ﻿Sample collection and fungal isolation

Dead wood samples were collected in Guiyang, Guizhou province, China, in September 2023. The specimens were taken to the laboratory in envelopes to observe and check their morphological characteristics. The samples were observed by a SMZ-T4 continuous zoom stereomicroscope (Optec stereomicroscope SMZ-T4, China), and the fruiting bodies on the samples were picked with needles and then made into slides to observe their morphological characteristics under the Zeiss microscope (Axioscope 5, China). Pure cultures were obtained by single spore isolation as described in Senanayake et al. (2020). After 2–3 days of spore germination, it was transferred to potato dextrose agar medium (PDA) for pure culture at 25 °C for 14 days. A Nikon digital camera (Canon 700D) was used to photograph the fruiting bodies on natural substrates. Zeiss Scope5 compound microscope Axioscope 5 (Carl Zeiss Microscopy GmbH, Jena, Germany) DIC and an axial camera were used to take photographs of microstructure. We measured 50 *conidia* and 10 *conidiogenous cells.* Dried holotype specimens were preserved in the herbarium of the Department of Plant Pathology, Agricultural College, Guizhou University (HGUP). Ex-type cultures were deposited in the Culture Collection at the Department of Plant Pathology, Agriculture College, Guizhou University, P.R. China (GUCC). Taxonomic information of the new species was submitted to MycoBank (www.mycobank.org), and accession numbers were provided in the taxonomy section of this paper.

### ﻿DNA extraction, amplification via PCR, and sequencing

Fungal DNA was extracted from the fresh mycelium grown on the PDA (c = 40.1 g/l) medium in an incubator at 25 °C for 30–40 days. Fresh mycelium was scraped using a sharpened plastic straw and placed into a 1.5 mL centrifuge tube. Then, it was ground into powder with a grinder. Genomic DNA was extracted following the instruction book of the Biospin Fungus Genomic DNA Extraction Kit (BioFlux®). The DNA was stored at −20 °C for long-term usage. Information on primers used for the amplification of internal transcribed spacers (ITS), small subunit rDNA (SSU), large subunit rDNA (LSU), translation elongation factor 1-α gene region (*ef1-α*), RNA polymerase II second largest subunit (*rpb2*), and beta-tubulin (*β-tubulin*) genes is presented in Table 1. The ampliﬁcation reactions were performed by following the protocol: 20 µL of PCR reaction mixture contained 1 µL of DNA template, 1 µL each of the forward and reverse primers (10 µm), 7 µL of double-distilled water (ddH_2_O), and 10 µL of 2Mix (Vazyme Biotech Co., Ltd). The PCR thermal cycling program conditions are presented in Table 1. PCR products were sequenced using Sanger sequencing conducted by Sangon Biotech (Shanghai) Co., Ltd. The PCR products were sequenced, and the sequence data were deposited in GenBank, as shown in Table 1 (Dai et al. 2017).

**Table 1.** The primers and polymerase chain reaction (PCR) thermal cycling programs for each locus.

Genes	Primers	PCR Thermal Cycle Protocols
ITS	ITS4	(94 °C, 30 s, 55 °C, 50 s, 72 °C, 1 min) 35 cycles
	ITS5	
LSU	LR0R	
	LR5	
SSU	NS1	
	NS4	
*ef1-α*	EF1-983F	
	EF1-2218R	
*rpb2*	fRPB2-5f	(94 °C, 1 min, 52 °C, 2 min, 72 °C, 90 s) 40 cycles
	fRPB2-7cr	
*β-tubulin*	Btub2Fd	(94 °C, 30 s, 58 °C, 30 s, 72 °C, 90 s) 35 cycles
	Btub4Rd	

### ﻿Phylogenetic analyses

Phylogenetic analysis was performed using DNA sequence data obtained from GenBank (https://www.ncbi.nlm.nih.gov/, accessed on 21 September 2024) (Table 2) and following previous publications (Dai et al. 2022; Wu et al. 2023). Sequence data were aligned using the online MAFFT v. 7.307 (http://mafft.cbrc.jp/alignment/server/, accessed on 21 September 2024; Katoh et al. 2019), and trim ambiguously aligned positions were done using trimAl (21 September 2024; Capella-Gutiérrez et al. 2009). The software BioEdit version 7.2.5 was utilized to make additional manual edits (Hall 1999). Ambiguous regions were excluded from the analysis, and gaps were treated as missing data. The aligned sequences that will be used for maximum likelihood (ML) were concatenated using Sequence Matrix v. 1.7.8 (Vaidya et al. 2011) and conducted with IQ-TREE (Nguyen et al. 2015; Trifinopoulos et al. 2016) via the IQ-TREE web server (accessed on 21 September 2024, http://iqtree.cibiv.univie.ac.at). The concatenated sequence alignments used for Bayesian inference analysis (BI) were acquired from MEGA versions 7.0.14 and 10.1.0, as reported by Kumar et al. (2016) and Tamura et al. (2021), respectively. Geneious Prime® 2023.2.1 Java Version 11.0.18+10 (64-bit) software (Biomatters Inc., Boston, USA) was used to convert the file format. Two approaches were applied to determine the phylogenetic relationships of the newly identified strains: maximum likelihood (ML) and Bayesian inference (BI) analyses. The final phylogenetic topologies were visualized with FigTree v.1.4.0 (http://tree.bio.ed.ac.uk/software/figtree/) and were modified in Microsoft Office PowerPoint 2021 (Microsoft, Redmond, USA).

**Table 2.** Species and GenBank accession numbers of DNA sequences used in the phylogenetic analysis.

Taxon	Strain*	Host	Origin	GenBank accession numbers				
				ITS	LSU	*rpb2*	*ef1-α*	*β-tubulin*
*Elongatopedicellatalignicola*	MFLUCC 15-0642	dead wood	Thailand	KX421369	KX421368	-	-	-
*Neoroussoellaalishanense*	FU31016 T	dead culms	China	MK503816	MK503822	MN037756	MK336181	-
*N.bambusae*	MFLUCC 11-0124 T	bamboo	Thailand	KJ474827	KJ474839	KJ474856	KJ474848	-
*N.chiangmaiensis*	MFLUCC 22-0168 T	dead culms	Thailand	NR189405	OQ065735	OQ186450	OQ186448	-
*N.clematidis*	MFLU 17-1467 T	*Clematissubumbellata*	Thailand	NR170813	-	-	-	-
	MFLUCC 17-2061	*Clematissubumbellata*	Thailand	MT310632	MT214587	MT394701	MT394645	-
*N.entadae*	MFLUCC 18-0243	*Leucaena* sp.	Thailand	MK347786	MK348004	MK434866	MK360065	-
	NI213	*Magnolia* sp.	Thailand	OL703580	OL457703		OM505027	
*N.fulvicomae*	MFLU 17-1471 T	*Clematisfulvicoma*	Thailand	NR170814	-	MT394702	MT394646	-
*N.heveae*	MFLUCC 17-2069	*Clematissubumbellata*	Thailand	MT310634	MT214589	MT394703	MT394647	-
*N.lenispora*	GZCC 16-0020 T	dead branch	China	NG068779	-	-	-	-
*N.leucaenae*	MFLUCC 17-0927 T	*Pterocarpus* sp.	Thailand	NR165226	-	MK434896	MK360066	-
	MFLUCC 18-1544	*Leucaena* sp.	Thailand	MK347767	MK347984	MK434876	MK360067	-
*Leucaenamagnoliae*	MFLU 18-1022	*Magnoliagrandiflora*	China	MK801232	MK801230		MK834373	
***N.guizhouensis***	**GUCC 24-0196**	**dead wood**	**China**	**PQ404885**	**PQ475844**	**PQ438559**	**PQ438562**	-
	**GUCC 24-0197 T**	**dead wood**	**China**	**PQ404886**	**PQ475842**	**PQ438557**	**PQ438560**	-
	**GUCC 24-0198**	**dead wood**	**China**	**PQ404887**	**PQ475843**	**PQ438558**	**PQ438561**	-
*N.lignicola*	MUT 5373 T	*Padinapavonica*	Italy	NR169908	MN556321	MN605916	MN605896	-
*N.peltophora*	MFLUCC 21-0071 T	*Peltophorumdubium*	Thailand	NG149003	MZ567206	MZ605442	MZ605441	-
*N.sedimenticola*	CGMCC 3 22468	*Sediment*	China	OQ798948	OQ758143	OQ809007	OQ809045	-
*N.solani*	CPC 26331 T	*Solanummauritianum*	France	NR145198	-	-	-	-
*N.thailandica*	NI258 T	*Magnolia* sp.	Thailand	OL703581	OL457704	ON502386	OM505028	-
*Nothoroussoellairregularis*	CGMCC 3.22466	sediment	China	NR191243	OQ758150	OQ809014	OQ809052	-
	CGMCC 3.22467	sediment	China	-	OQ758151	OQ809015	OQ809053	-
*Pseudoroussoellachromolaenae*	MFLUCC 17-1492 T	Dead aerial stems	Thailand	NR168861	MT214439	-	MT235769	-
	MFLUCC 17-2062	*Clematissubumbellata*	Thailand	MT310635	MT214590	MT394704	MT394648	-
*P.elaeicola*	MFLUCC 17-2059	*Clematissubumbellata*	Thailand	-	MT214591	MT394705	-	-
	MFLUCC:17-1483	*Chromolaenaodorata*	Thailand	MT214348	MT214442	MT235808	MT235772	-
*Roussoellaangusta*	MFLUCC 15-0186 T	*bamboo*	Thailand	-	KT281979	-	-	-
*R.aquatica*	MFLUCC 18-1040 T	submerged wood	China	NR171975	NG073797	-	-	-
*R.chiangraina*	MFLUCC 10-0556 T	bamboo	Thailand	KJ474828	KJ474840	KJ474857	KJ474849	-
*R.doimaesalongensis*	MFLUCC 14-0584 T	bamboo	Thailand	KY026584	KY000659	KY678394	KY651249	-
*R.guttulata*	MFLUCC 20-0102 T	bamboo	Thailand	NR172428	NG075383	MW022187	MW022188	-
***R.guizhouensis***	**GUCC 24-0199 T**	**bamboo**	**China**	**PQ404882**	**PQ475845**	**PQ399769**	**PQ438563**	-
	**GUCC 24-0200**	**bamboo**	**China**	**PQ404883**	**PQ475847**	**PQ399770**	**PQ438564**	-
	**GUCC 24-0201**	**bamboo**	**China**	**PQ404884**	**PQ475846**	**PQ399771**	**PQ438565**	-
*R.hysterioides*	CBS 546.94	-	France	KF443405	KF443381	KF443392	KF443399	-
*R.intermedia*	NBRC 106245	*Sasakurilensis*	Japan	KJ474831	AB524624	-	-	-
*R.japanensis*	MAFF 239636 T	*Sasaveitchii*	Japan	KJ474829	AB524621	AB539101	AB539114	-
	GMBCC1117	*bamboo*	China	OM891811	OM884023	ON098382	ON098345	-
*R.kunmingensis*	KUMCC 18-0128 T	*bamboo*	China	MH453491	MH453487	MH453484	MH453480	-
	GMBCC1057	*bamboo*	China	OM891798	OM884014	ON098362	ON098354	-
*R.magnatum*	MFLUCC 15-0185 T	*bamboo*	China	-	KT281980	-	-	-
*R.margidorensis*	MUT 5329 T	*Padinapavonica*	Italy	KU314944	MN556322	MN605917	MN605897	-
*R.mediterranea*	MUT 5369 T	*Padinapavonica*	Italy	KU314947	MN556324	MN605919	MN605899	-
*R.multiloculate*	GMBCC1056 T	bamboo	China	OM891799	OM884015	ON098369	ON098343	-
	GMBCC1071	bamboo	China	ON159383	OM755586	ON098368	ON098342	-
*R.neopustulans*	MFLUCC 11-0609 T	bamboo	Thailand	KJ474833	KJ474841	-	KJ474850	-
	MFLUCC 12-0003	bamboo	Thailand	KU940130	KU863119	-	-	-
*R.nitidula*	MFLUCC 11-0182 T	*Bambusa*	Thailand	KJ474835	KJ474843	KJ474859	KJ474852	-
	MFLUCC 11-0634	bamboo	Thailand	KJ474834	KJ474842	KJ474858	KJ474851	-
*R.padinae*	MUT 5503 T	*Padinapavonica*	Italy	KU158170	MN556327	MN605922	MN605902	-
	GMBCC1126	bamboo	China	OM891816	OM884025	ON098383	ON098356	-
	GMBCC1121 T	bamboo	China	OM891814	OM755608	ON098378	ON098346	-
	IFRDCC 3103	bamboo	China	ON228188	ON228184	ON244450	ON244452	-
*R.pseudohysterioides*	MFLUCC 13-0852 T	bamboo	Thailand	KU940131	KU863120	-	KU940198	-
	KUMCC 18-0111	bamboo	China	MH453490	MH453486	MH453483	MH453479	-
*R.pustulans*	MAFF 239637 T	*Sasakurilensis*	Japan	KJ474830	AB524623	AB539103	AB539116	-
*R.scabrispora*	MFLUCC 11-0624 T	bamboo	Thailand	KJ474836	KJ474844	KJ474860	KJ474853	-
*R.scabrispora*	GMBCC1104	bamboo	China	OM891807	OM755616	ON098373	ON098349	-
*R.siamensis*	MFLUCC 11-0149 T	Bambusa	Thailand	KJ474837	KJ474845	KJ474861	KJ474854	-
	GMBCC1119 T	bamboo	China	OM891813	OM884024	ON098379	ON098357	-
	IFRDCC 3101	bamboo	China	ON228187	ON228183	ON244451	ON244453	-
*R.thailandica*	MFLUCC 11-0621 T	bamboo	Thailand	KJ474838	KJ474846	-	-	-
*R.tuberculata*	MFLUCC 13-0854 T	bamboo	Thailand	KU940132	KU863121	-	KU940199	-
	GMBCC1123	bamboo	China	OM891815	OM755613	ON098380	ON098352	-
*R.uniloculata*	GMBCC1110 T	bamboo	China	OM891809	OM801286	ON098374	ON098360	-
	DDQ01005-2	bamboo	China	OM891817	OM884026	ON098375	ON098361	-
*R.verrucispora*	CBS 125434 T	*Sasakurilensis*	Japan	KJ474832	AB524622	AB539102	AB539115	-
*R.yunnanensis*	KUMCC 18-0115 T	bamboo	China	MH453492	MH453488	-	MH453481	-
*Roussoellopsismacrospora*	MFLUCC 12-0005 T	bamboo	Thailand	KJ739604	KJ474847	KJ474862	KJ474855	-
*R..tosaensis*	KT 1659	bamboo	Japan	-	AB524625	AB539104	AB539117	-
*Setoarthopyreniachromolaenae*	MFLUCC 17-1444 T	*Chromolaenaodorata*	Thailand	NG070153	MT214438	MT235805	MT235768	-
*Xenoroussoellatriseptata*	MFLUCC 17-1438 T	*Chromolaenaodorata*	Thailand	MT214343	MT214437	MT235804	MT235767	-
	KNUF-20-NI009	Soil	South Korea	LC719282	LC719283	LC723532	LC723531	-
	**GUCC 24-0202**	**dead wood**	**China**	**PQ404879**	**PQ475839**	**PQ399766**	**PQ438566**	-
	**GUCC 24-0203**	**dead wood**	**China**	**PQ404880**	**PQ475840**	**PQ399767**	**PQ438567**	-
	**GUCC 24-0204**	**dead wood**	**China**	**PQ404881**	**PQ475841**	**PQ399768**	**PQ438568**	-
*Versicolorisporiumtriseptatum*	JCM 14775 T	*Pleioblastuschino*	Japan	AB365596	AB330081	-	-	-
	NMX1222	Bamboo	China	OL741378	OL741318	-	-	-
*Xenodidymellaapplanata*	CBS 205.63	*Rubusidaeus*	Netherlands	GU237798	-	KP330402	-	GU237556
	CBS 195.36 T	*Rubusidaeus*	Netherlands	KT389548	-	MT018280	-	KT389852
*Xenodid.asphodeli*	CBS 375.62 T	*Asphodelusalbus*	France	KT389549	-	KT389689	-	MT005716
	CBS 499.72	*Asphodelusramosus*	Italy	KT389550	-	MT018282	-	KT389853
*Xenod.camporesii*	MFLUCC 17-2309 T	*Dipsacus* sp.	Italy	NR169976	-	-	-	MN871955
*Xenod.catariae*	CBS 102635	*Nepetacatenaria*	Netherlands	GU237727	-	KP330404	-	GU237524
*Xenodid.clematidis*	MFLUCC 16-1365 T	*Clematisvitalba*	Italy	MT310600	-	-	-	-
*Xenodid.glycyrrhizicola*	IRAN 4162C;, SCUA-Ahm-S45	*Ziziphusmauritiana*	Iran	MZ145256	-	MZ169396	-	MZ169403
*Xenod.glycyrrhizicola*	CBS 684.97T	*Glycyrrhizalepidota*	New Zealand	MN973606	-	MT018283	-	MT005717
***Xenod.guizhouensis***	**GUCC 24-0205 T**	**dead wood**	**China**	**PQ404876**	-	**PQ399763**	-	**PQ399760**
	**GUCC 24-0206**	**dead wood**	**China**	**PQ404877**	-	**PQ399764**	-	**PQ399761**
	**GUCC 24-0207**	**dead wood**	**China**	**PQ404878**	-	**PQ399765**	-	**PQ399762**
*Xenod.humicola*	CBS 220.85	*Franseria* sp.	U.S.A.	GU237800	-	KP330422	-	GU237617
*Xenod.iranica*	SCUA-Ahm-Sh1-2	*Callistemoncitrinus*	Iran	MZ145253	-	-	-	MZ169400
	IRAN 4142CT;, SCUA-Saf-Sh1	*Callistemoncitrinus*	Iran	MZ145252	-	-	-	MZ169399
*Xenod.menthae*	SCUA-Ahm-W4	Mentha×piperita	Iran	OK257018	-	OK247739	-	OK247745
	SCUA-Ah-W4-2	Mentha×piperita	Iran	OK257019	-	OK247740	-	OK247746
*Xenod.saxea*	CBS 419.92T	Corroded mediterranean marble	Germany	GU237860	-	KP330429	-	GU237655
*Xenodidymella* sp.	CBS 141233	*Anchusaitalica*	Iran	MN973608	-	MT018285	-	MT005719
*Xenod.weymaniae*	CBS 144960 T	-	Netherlands	MN823588	-	MN824613	-	MN824762
*Neod.xanthina*	CBS:383.68 T	*Delphinium* sp.	Netherlands	NR135994	-	KP330431	-	GU237668
	CBS:168.70	*Delphinium* sp.	Netherlands	KT389533	-	MT018290	-	KT389831
*Didymellasubrosea*	CBS 733.79 T	*Abiesalba*	France	NR170787	-	MT018174	-	MT005643
*D.subglobispora*	CBS 364.91	*Ananassativus*	-	MN973531	-	MT018153	-	MT005634

*T = Type. The strains in this study are in bold. CBS: Culture collection of the Westerdijk Fungal Biodiversity Institute, Utrecht, The Netherlands; CPC: Culture collection of Pedro Crous, housed at the Westerdijk Institute; GUCC: Herbarium of the Department of Plant Pathology, Agricultural College, Guizhou University, China; MFLUCC: Mae Fah Luang University Culture Collection, Thailand.

## ﻿Results

### ﻿Phylogenetic analyses

**Figure 1.**
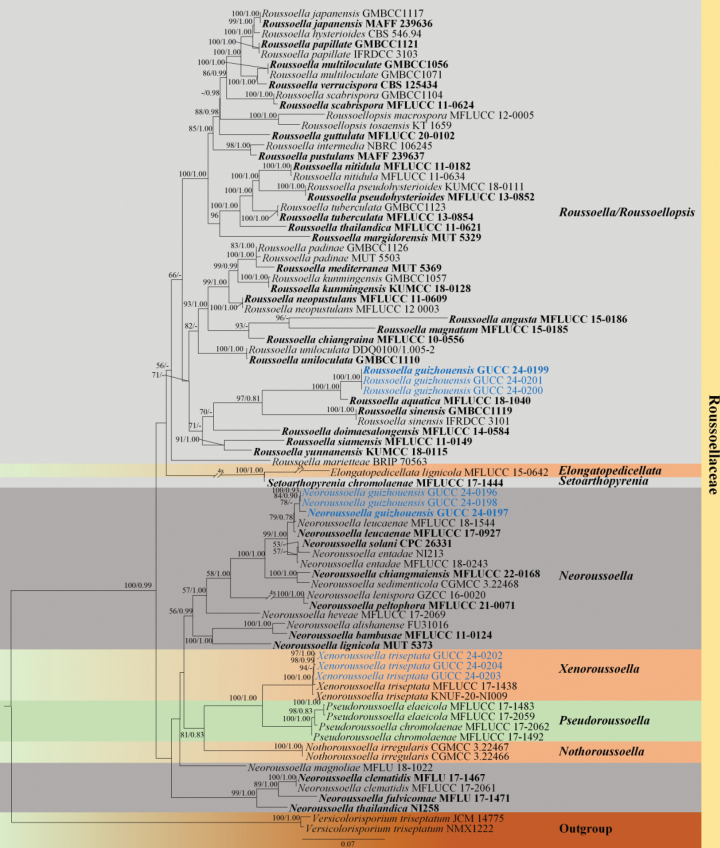
Phylogenetic tree generated from maximum likelihood analysis based on combined ITS, LSU, *rpb2*, and *ef1-α* sequence data for 79 strains of Roussoellaceae. This tree is rooted with two strains of *Versicolorisporiumtriseptatum* (JCM 14775 and NMX1222). Maximum likelihood bootstrap values ≥ 50% and Bayesian posterior probabilities ≥ 0.80 (MLBS/BIPP) are given at the nodes. The species obtained in this study are blue, and the ex-type taxa are bold. Bar = 0.07 represents the estimated number of nucleotide site substitutions per branch. The second phylogenetic tree (Fig. 2) was constructed based on a comprehensive polygenic analysis of the ITS, *rpb2*, and *β-tubulin* gene regions, utilizing a dataset that included 22 strains of *Xenodidymella*, with two outgroup strains, *Didymellasubrosea* (CBS 733.79) and *D.subglobispora* (CBS 364.91). The final alignment, which accounted for gaps, comprised 1,412 characters (ITS: 1–484, *rpb2*: 485–1079, *β-tubulin*: 1080–1412). These datasets were then used to perform maximum likelihood (ML) and Bayesian inference (BI) analyses. The ML tree was the primary representation for illustrating the phylogenetic relationships among the diverse taxa within *Xenodidymella*. This tree elucidated the evolutionary positioning and interrelationships of *Xenodidymella* and its related species. Notably, our strains (GUCC 24-0205, GUCC 24-0206, and GUCC 24-0207) formed an obviously distinct lineage, separate from other *Xenodidymella* species, with high support by 100% MLB and 1.00 BIPP.

The phylogenetic analyses (Fig. 1) utilized a comprehensive dataset comprising four loci (ITS, LSU, *rpb2*, and *ef1-α*), including 79 strains of Roussoellaceae species, with two outgroup strains, *Versicolorisporiumtriseptatum* (strain JCM 14775) and *V.triseptatum* (strain NMX1222). The final alignment comprised 3,026 characters (ITS: 1–425, LSU: 426–1210, *rpb2*: 1211–2233, and *ef1-α*: 2234–3026), including gaps. Both maximum likelihood (ML) and Bayesian inference (BI) methods were conducted. The ML tree was selected to represent the phylogenetic relationships of Roussoellaceae taxa. The analyses revealed that the newly isolated strains (GUCC 24-0197, GUCC 24-0196, and GUCC 24-0198) formed a distinct branch with strong statistical support (MLBS 81%/BIPP < 0.80). This clade emerged as a sister group to *Neoroussoellaleucaenae* (MFLUCC 18-1544), supported by moderate statistical values (78% MLBS/0.69 BIPP). Similarly, strains GUCC 24-0199, GUCC 24-0200, and GUCC 24-0201 formed a distinct branch, with robust support (100% MLBS and 1.00 BIPP). This clade was positioned as a sister group to *R.aquatica* (MFLUCC 18-1040), with exhibited high support (100% MLBS/1.00 BIPP). The strains GUCC 24-0202, GUCC 24-0203, and GUCC 24-0204 clustered with two strains of *Xenoroussoellatriseptata* (MFLUCC 17-1438 and KNUF-20-NI009), which demonstrated 100% MLBS and 1.00 BIPP support.

### ﻿Taxonomy

#### ﻿*Neoroussoella* Jian K. Liu, Phook. & K.D. Hyde, *Phytotaxa* 181(1): 21 (2014)

*Neoroussoella* (Roussoellaceae) was introduced by Liu et al. (2014) to accommodate species that exhibited morphological characteristics distinct from other genera. *Neoroussoella* spp. are typically saprobic, inhabiting decaying wood and plant material, and have been found primarily in subtropical and tropical regions. Morphologically, the genus is characterized by immersed to semi-immersed ascomata, cylindrical to clavate asci, and hyaline, septate ascospores (Pem et al. 2024). Phylogenetic analyses based on multilocus DNA sequencing have further supported the placement of *Neoroussoella* as a distinct genus within Roussoellaceae.

##### ﻿ Neoroussoella guizhouensis

Taxon classificationFungiPleosporalesRoussoellaceae

S.Q. Guo, K.D. Hyde & Yong Wang bis sp. nov.

C7A0504F-D6C1-5C06-84C0-347B53F54518

856167

Fig. 3

###### Etymology.

The name refers to Guizhou Province, where the fungus was collected.

###### Type.

China • Guizhou Province, Guiyang City, 26°6'N, 106°7'E, 1071 m, 7 September 2023, from dead wood culms, coll. S.Q. Guo, HGUP 24-0071 (holotype), ex-type culture GUCC 24-0197 (ITS: PQ404886, LSU: PP949847, *rpb2*: PQ438557, *ef1-α*: PQ438560).

###### Description.

***Saprobic*** on dead wood culms. ***Sexual morph***: undetermined. ***Asexual morph***: On the natural substrate: ***Pycnidia*** 81–137 × 65–1198 µm (x̄ = 108.5 × 81.9 µm, n = 10), ellipsoidal to nearly circular, inlaid on the surface of the substrate with a black ring at the edge, transparent in the center, and smooth in surface texture. On PDA: ***Conidiophores*** reduced to ***conidiogenous cells*. *Conidiogenous cell*** 9–35 × 1–6 µm (x̄ = 20 × 3.8 µm, n = 10), enteroblastic, indeterminate, discrete, unbranched, clavate or ampulliform to lageniform, hyaline, aseptate, smooth-walled. ***Conidia*** (2.9–)3.4–3.5(–4) × (2–)2.4–2.5(–2.6) µm (x̄ = 3.5 × 2.5 µm, n = 50), oblong to ellipsoid, with rounded to obtuse ends, hyaline, aseptate, smooth-walled.

###### Culture characteristics.

Under dark conditions at 25 °C, the colony diameter can reach 80 mm within seven days on PDA medium. Center of the colony is light gray, accompanied by a white ring. Hyphae at the edge are sparse, slightly irregular, and lace-like, and several vertical stripes spread out at the center. Reverse side is black to light red, and the edge is yellowish.

###### Material examined.

China • Guizhou Province, Guiyang City, 26°6'N, 106°7'E, 1071 m, 7 September 2023, from dead wood culms, coll. J.E. Sun, HGUP 24-0071 (***holotype***), living culture GUCC 24-0197, GUCC 24-0196, and GUCC 24-0198.

###### Notes.

*Neoroussoellaguizhouensis* is a saprobic fungus found on dead wood culms. According to the multi-gene phylogenetic analysis (Fig. 1), our isolates formed a distinct clade, sister to *Neoroussoellaleucaenae*, which was previously reported from decaying pods of *Leucaena* sp. and *Pterocarpus* sp. in Thailand (Jayasiri et al. 2019). This relationship is supported by statistical values of 79% MLBS and 0.78 BIPP (Fig. 1). On PDA, colonies of *N.guizhouensis* display a light gray center surrounded by a white ring, distinguishing it from *N.solani*, which exhibits white to lavender-gray colonies and produces rosy buff to grayish-rose pigments (Mochizuki et al. 2017). Morphological data for *N.leucaenae* and *N.entadae* on PDA are unavailable in the literature. However, on MEA, *N.leucaenae* shows gray to brown hyphal growth with a radially arranged brown edge and a two-layered reverse side, dark brown and brown. In contrast, *N.entadae* displays irregular edges with a thin, light-colored outer layer embedded in the medium, a three-layered reverse side of dark brown with distinct boundaries, and scattered pycnidia throughout the culture medium (Jayasiri et al. 2019). Phylogenetically, the new species was grouped with *N.guizhouensis*, *N.leucaenae*, *N.solani*, and *N.entadae*. However, the morphological distinctions are evident among those species, particularly in the conidiogenous cells. Although all species exhibit bottle-shaped sporogenic structures, *N.leucaenae* is characterized by flask- to cylindrical-shaped cells with a thicker neck and nearly round base. In contrast, *N.guizhouensis* has a narrow-necked bottle shape, whereas *N.solani* shows a broader base, with some cells appearing nearly oval. *N.entadae* displays phialidic, ampulliform to cylindrical conidiogenous cells. The dimensions of the conidiogenous cells differ significantly among the species: *N.guizhouensis* (9–35 × 1–6 µm), *N.leucaenae* (5.5–9 × 3–4 µm), *N.solani* (5–10 × 3.5–5 µm), and *N.entadae* (3.5–5.6 × 0.7–1.8 µm). Conidia size comparisons also highlight differences: *N.guizhouensis* (2.9–4 × 2–2.6 µm), *N.leucaenae* (3.5–4.5 × 1.9–2.6 µm), *N.solani* (3.9–5.3 × 1.9–2.2 µm), and *N.entadae* (3–4 × 1.7–1.9 µm). While *N.guizhouensis* and *N.leucaenae* are similar in spore size, they have shorter lengths and narrower widths compared to *N.solani* and exhibit similar lengths but narrower widths compared to *N.entadae*. Additionally, nucleotide sequence comparisons of the *ef1-α* and *rpb2* loci revealed differences between *N.guizhouensis* (GUCC 24-0197) and *N.leucaenae* (MFLUCC 17-0927), with 2-bp and 16-bp discrepancies, respectively. Based on these findings, *N.guizhouensis* is established as a new species.

#### ﻿*Roussoella* Sacc., Atti Inst. Veneto Sci. lett., ed Arti, Sér. 6 6: 410 (1888)

*Roussoella* was introduced by Saccardo and Paoletti (1888) and subsequently designated as the type genus of Roussoellaceae by Liu et al. (2014). *Roussoella* species are predominantly saprobic, commonly found on terrestrial or aquatic flowering plants and on submerged wood in freshwater habitats, with a particular affinity for bamboo and palm trees (Dong et al. 2020). Morphologically, the sexual state of *Roussoella* is characterized by immersed apothecia, cylindrical asci, and spindle-shaped ascospores (Hyde et al. 2013; Liu et al. 2014; Dai et al. 2022), while the asexual state is distinguished by ellipsoidal or ampulliform conidia (Dai et al. 2017; Jiang et al. 2019; Dong et al. 2020).

##### ﻿ Roussoella guizhouensis

Taxon classificationFungiPleosporalesRoussoellaceae

S. Q. Guo, K.D. Hyde & Yong Wang bis sp. nov.

1323726E-3A84-5B0E-93CE-4C6D42253D16

856168

Fig. 4

###### Etymology.

The name refers to Guizhou Province, where the fungus was collected.

###### Type.

China • Guizhou Province, Guiyang City, 26°6'N, 106°7'E, 1071 m, 24 September 2023, from dead bamboo culms, coll. J.F. Mo, HGUP 24-0072 (holotype), ex-type culture GUCC 24-0199 (ITS: PQ404882, LSU: PP949847, *rpb2*: PQ399769, *ef1-α*: PQ438563).

###### Description.

***Saprobic*** on dead bamboo culms. ***Sexual morph***: undetermined. ***Asexual morph*: *Pycnidia*** 998–3121 × 143–853 µm (x̄ = 1749.6 × 430.9 µm, n = 10), stromatic on the substrate, develops above the epidermis and becomes raised when mature, ellipsoidal to irregular in shape, with a rough surface ranging in color from reddish-brown to black, composed of numerous dense, glossy black ***stromata*** globules stacked layer upon layer. ***Stroma*** ranges in color from light brown to black, being transparent and shaped like a hemisphere to a near-perfect sphere, with uniform size, smooth surface, glossy, with half embedded in the natural substrate and the other half exposed, a circular aperture is present at the top. On PDA medium: ***Pycnidia*** superficial, float above the ***stromata***, shaped like hemispheres or spheres, appearing as viscous, mucoid masses with a flat base, colour ranging from dark brown to black, lacking a pycnidial wall, release brownish-yellow ***conidia*** mass. ***Conidiophores*** reduced to ***conidiogenous cells***. ***Conidiogenous cell*** 5–8 × 1–2 µm (x̄ = 6.2 × 1.7 µm, n = 10), enteroblastic, indeterminate, discrete, unbranched, clavate or ampulliform to lageniform, transparent to light brown, aseptate, smooth-walled. ***Conidia*** (1.7–)2.2–2.3(–2.6) × (1.3–)1.5–1.6(–1.9) µm (x̄ = 2.3 × 1.6 µm, n = 50), oblong to ellipsoid, with rounded to obtuse ends, light brown to transparent, smooth-walled.

###### Culture characteristics.

Under dark conditions at 25 °C, the colony reaches a diameter of 90 mm within seven days on PDA medium. Colony is dense, exhibiting a rough surface and a regular shape with a fluffy margin. Center is velvety, characterized by a grayish-white coloration surrounded by gray margins. Reverse side is grayish-black, featuring a small grayish-white circle in the middle, which produces a black pigment.

###### Material examined.

China • Guizhou Province, Guiyang City, 26°6'N, 106°7'E, 1071 m, 24 September 2023, from dead bamboo culms, coll. J.F. Mo, HGUP 24-0072 (***holotype***), living culture GUCC 24-0199, GUCC 24-0200 and GUCC 24-0201.

###### Notes.

Based on the multigene phylogenetic tree (Fig. 1), our strains grouped into a distinct clade, forming a sister clade to *Roussoellaaquatica* (MFLUCC 18-1040, Dong et al. 2020), found in submerged wood from a stream in Yunnan, China, with 100 MLBS/BIPP 1.00 statistical support. A comparison of nucleotide data within ITS and LSU loci revealed discrepancies between *R.guizhouensis* and *R.aquatica* (ITS: 16 bp and LSU: 7 bp). Morphologically, *R.guizhouensis* is distinguishable from *R.aquatica* by having larger conidiogenous cells (5–8 × 1–2 µm *vs.* 3–4 × 1.5–2 µm) and smaller conidia (1.8–2.7 × 1.3–1.9 µm *vs.* 2.7–3.5 × 2–2.5 µm). Thus, we introduce *Roussoellaguizhouensis* as a new taxon.

#### ﻿*Xenodidymella* Qian Chen & L. Cai, Stud. Mycol. 82: 205 (2015)

*Xenodidymella* was established based on the type species, *X.applanata*, along with three additional species: *X.asphodeli*, *X.catariae*, and *X.humicola*. Phylogenetic analyses based on multilocus data have demonstrated that these species formed a monophyletic lineage within phoma-like taxa (Chen et al. 2015). Most species of *Xenodidymella* are recognized as plant pathogens, capable of causing various diseases in crops and plants worldwide (Boerema et al. 2004). Additionally, several *Xenodidymella* species function as saprobes, inhabiting living and dead plant tissues (Hyde et al. 2020; Phukhamsakda et al. 2020).

##### ﻿ Xenodidymella guizhouensis

Taxon classificationFungiPleosporalesDidymellaceae

S.Q. Guo, K.D. Hyde & Yong Wang bis sp. nov.

D8BEE9FF-E66A-5B49-A48E-5E627FFB99A0

856169

Fig. 5

###### Etymology.

The name refers to Guizhou Province, where the fungus was collected.

###### Type.

China • Guizhou Province, Guiyang City, 26°6'N, 106°6'E, 1071 m, 7 September 2023, from dead wood culms, coll. S.M. Fu, HGUP 24-0073 (holotype), ex-type culture GUCC 24-0205 (ITS: PQ404876, *rpb2*: PQ399763, *β-tubulin*: PQ399760).

###### Description.

***Saprobic*** on dead wood culms. ***Sexual morph***: undetermined. ***Asexual morph*: *Stromata*** grow above the epidermis and become hemispherically raised when mature. ***Stromata*** 61–587 µm high, 67–489 µm wide, fattened at the base, those ***stromata*** distributed singly, in groups, or scattered, and their color ranges from dark brown to black. On PDA medium, grows on epidermis and bulges when matures. ***Conidiophores*** degenerated into sporogenous cells. ***Conidiogenous cells*** 8–17 × 1–5 µm (x̄ = 17.9 × 2.6 µm, n = 10) wide, phialidic, ampulliform, or lageniform, hyaline, smooth-walled, and have two types of spore-producing structures: one type, long, thin tentacles that extend irregularly outward, other type, short and wide, and twisted together to form knots, and the ends of both rounded. ***Conidia*** (1.7–)2.7–2.8(–3) × (0.7–)0.9–1(–1.5) µm (x̄ = 2.5 × 1 µm, n = 50) oblong, hyaline, aseptate, with two small polar guttules at both ends, smooth, thin-walled.

###### Culture characteristics.

***Colonies*** on PDA reached a diameter of 20 mm after seven days at 25 °C. Hyphae white at the center, radiating outward from a pale-yellow inner ring to a light yellowish-brown periphery with a milky white outer ring. The colony reverse exhibited a dark yellow to bright yellow pigmentation.

Colonies on OA reached 32 mm after seven days at 25 °C. Initially, it was a small circle formed by uniform and sparse white hyphae. After 14 days, it grew into slightly sparse hyphae with white clutter inside and dense white hyphae at the edge in a slightly regular circle. On the surface, hyphae balls with uneven sizes from orange to white and tiny black spots were scattered, and sporogenous structures could be observed.

###### Material examined.

China • Guizhou Province, Guiyang City, 26°6'N, 106°6'E, 1071 m, 7 September 2023, from dead wood culms, coll. S.M. Fu, HGUP 24-0073 (holotype), living culture GUCC 24-0205, GUCC 24-0206, and GUCC 24-0207.

###### Notes.

**Figure 2.**
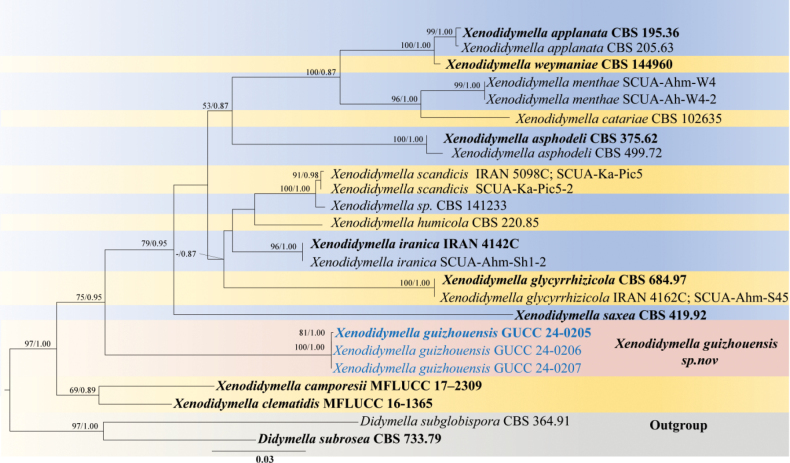
The phylogenetic tree generated from maximum likelihood analysis based on combined ITS, *β-tubulin*, and *rpb2* sequence data for 22 strains of *Xenodidymella*. This tree is rooted with *Didymellasubrosea* (CBS 733.79) and *D.subglobispora* (CBS 364.91). Maximum likelihood bootstrap values ≥ 50% and Bayesian posterior probabilities ≥ 0.80 (MLBS/BIPP) are given at the nodes. The species obtained in this study are blue, and the ex-type taxa are bold. Bar: 0.03 represents the estimated number of nucleotide site substitutions per branch.

Phylogenetic analyses (Fig. 2) revealed that the three isolates (GUCC 24-0205, GUCC 24-0206, and GUCC 24-0207) formed an independent branch. In morphology, they can be distinguished from *Xenod.clematidis* by their smaller conidiogenous cells (10–17(–20) × 1–2 µm *vs.* 2–6(–12) × 2.3–3.3 µm), although the conidiogenous cell size for *Xenod.saxea* remains undetermined. Additionally, *Xenod.guizhouensis* produces smaller *conidia* than both *Xenod.clematidis* and *Xenod.saxea* (2.9–4.3 × 1.7–2.4 µm *vs.* 4–8 × 2–5 µm and 3.5–7.5 × 2.5–4 µm), respectively.

**Figure 3.**
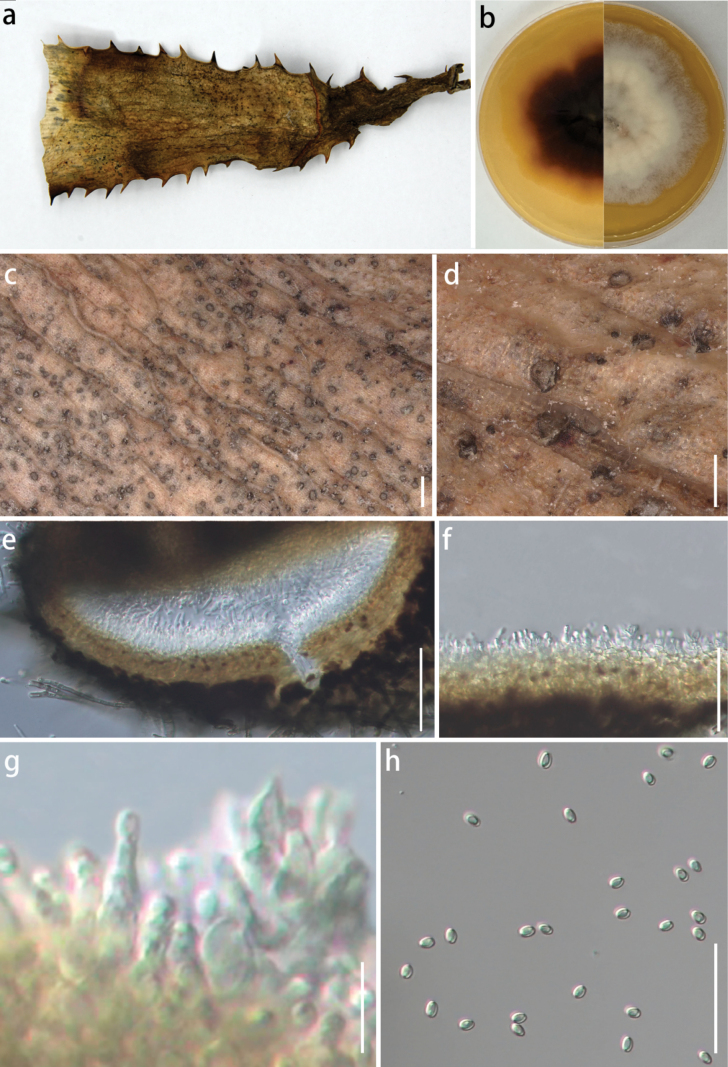
*Neoroussoellaguizhouensis* (GUCC 24-0197, holotype) **a** appearance on host surface **b** cultures on PDA from above and below **c, d** black ascostromata on host surface **e–g** conidiophores, conidiogenous cells, and conidia (h) conidia. Scale bars: 1000 µm (**c**); 250 µm (**d**); 50 µm (**e, f**); 12.5 µm (**g**); 25 µm (**h**).

**Figure 4.**
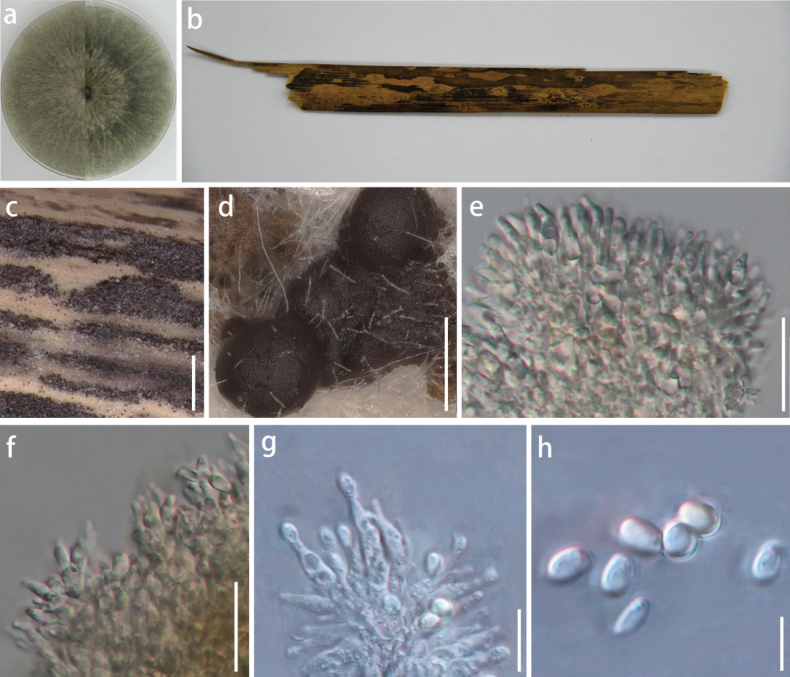
*Roussoellaguizhouensis* (GUCC 24-0199, holotype) **a** cultures on PDA from above and below **b** bamboo specimen **c, d** black conidiostromata on host surface **e–g** conidiophores, conidiogenous cells, and conidia **h** conidia. Scale bars: 500 µm (**c**); 200 µm (**d**); 25 µm (**e, f**); 15 µm (**g**); 2.5 µm (**h**).

**Figure 5.**
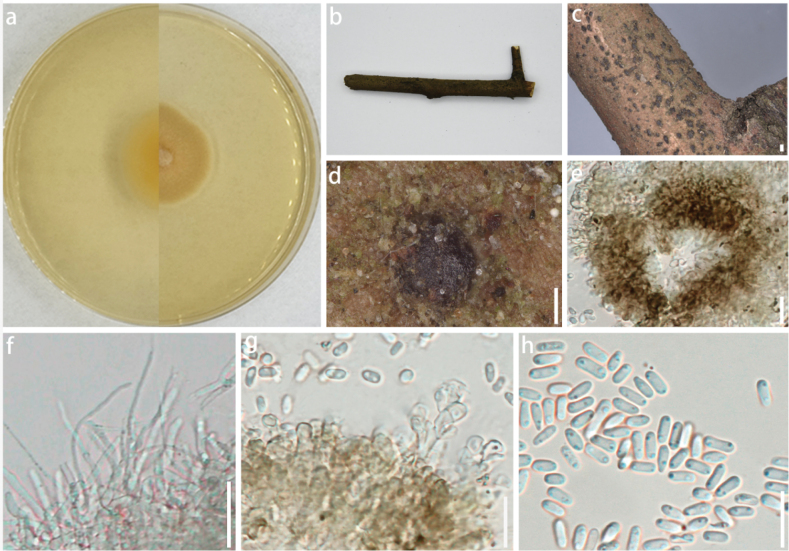
*Xenodidymellaguizhouensis* (GUCC 24-0205, holotype) **a** cultures on PDA from above and below **b** appearance on host surface **c, d** black *ascostromata* on host surface **e–h***conidiophores*, *conidiogenous cells*, *conidia*. Scale bars: 500 µm (**c**); 100 µm (**d**); 2.5 µm (**e**); 12.5 µm (**f**); 5 µm (**g**); 2.5 µm (**h**).

#### ﻿*Xenoroussoella* Mapook & K.D. Hyde, Fungal Diversity 101: 93 (2020)

*Xenoroussoella* is a genus within Roussoellaceae, classified under Pleosporales (Mapook et al. 2020). This genus comprises a group of fungi notable for their ecological roles as saprobes and plant pathogens. Recent phylogenetic studies have further elucidated the relationships within this genus, demonstrating its distinct placement within Roussoellaceae and highlighting its diversity (Mapook et al. 2020; Ryu et al. 2022).

##### ﻿ Xenoroussoella triseptata

Taxon classificationFungiPleosporalesDidymellaceae

Mapook & K.D. Hyde, Fungal Diversity 101: 93 (2020)

C58F1021-D49D-5412-A5C4-F65CCF9D4985

Fig. 6

###### Description.

***Saprobic*** on dead wood culms. ***Sexual morph***: see Mapook et al. (2020). ***Asexual morph*: *Pycnidia*** on natural host, stromatic, developing above the epidermis and becoming raised when mature. ***Stromata*** 60–786 × 43–306 µm (x̄ = 320.8 × 172.0 µm, n = 10), with a color ranging from dark brown to black, rough surface with tiny protrusions, some exhibit a certain degree of gloss. and exhibit circular, oval, or irregular shapes, adhering tightly to the surface of decaying wood. On PDA medium, ***Conidiophores*** reduced to ***conidiogenous cells*. *Conidiogenous cell*** 8–17 × 2–5 µm (x̄ = 11.5 × 3 µm, n = 10), enteroblastic, indeterminate, discrete, unbranched, clavate or ampulliform, aseptate, hyaline, smooth-walled. ***Conidia*** (3.3–) 3.7–3.8 (–4.7) × (2.8–) 3.1–3.2 (3.6) µm (x̄ = 3.8 × 3.2 µm, n = 50), oblong to ellipsoid or rounded to obtuse ends, aseptate, hyaline to pale yellow, smooth-walled.

###### Culture characteristics.

Under dark conditions at 25 °C, the colony reaches a diameter of 15 mm within seven days on PDA medium. The colony is dense, smooth, and irregularly shaped, with a lace-like margin and compact structure. The center is grayish-white, bordered by a white lace-like margin, while the outer edge is grayish-black. The reverse side of the colony is black, producing a dark pigment.

**Figure 6.**
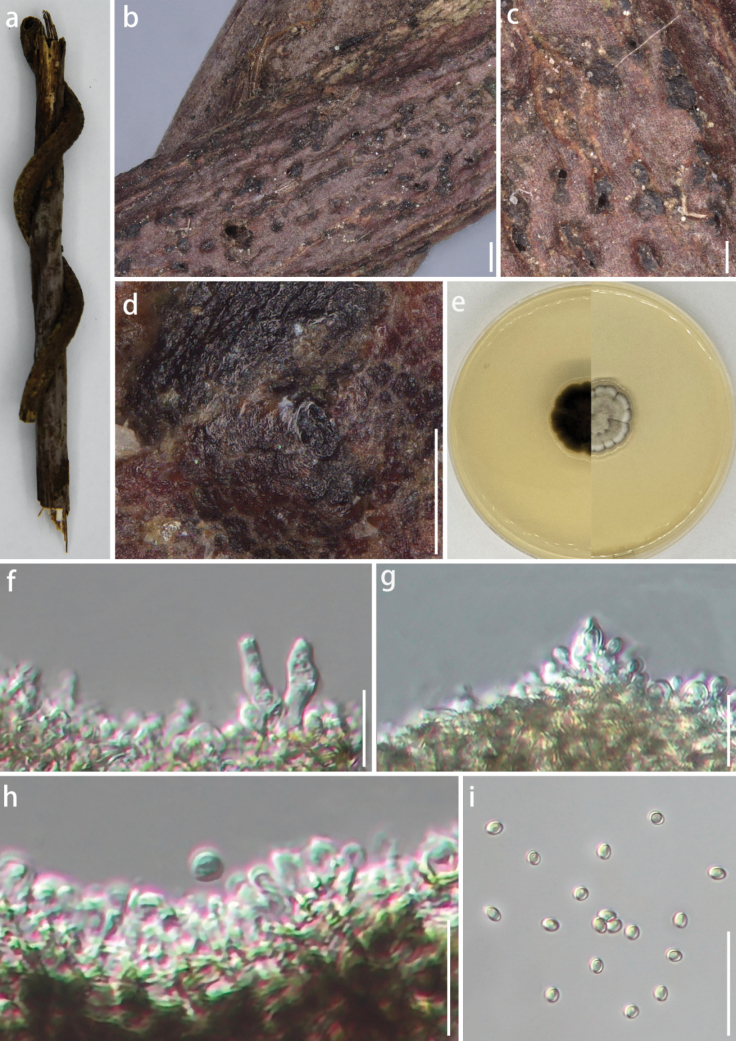
*Xenoroussoellatriseptata* (GUCC 24-0202, new geographical record) **a** appearance on host surface **e** cultures on PDA from above and below **b, c, d** black ascostromata on host surface **f–h** conidiophores, conidiogenous cells, and conidia (i) conidia. Scale bars: 500 µm (**b**); 250 µm (**c**); 100 µm (**d**); 12.5 µm (**f–h**); 25 µm (**i**).

###### Distribution.

China, Korea, Thailand.

###### Material examined.

China • Guizhou Province, Guiyang City, 26°6'N, 106°73'E, 1071 m, 7 September 2023, from dead wood culms, coll. J.E. Sun, HGUP 24-0074 (holotype), ex-type: GUCC 24-0202 (ITS: PQ404879, LSU: PQ475839, *rpb2*: PQ399766, *ef1-α*: PQ438566), living culture: GUCC 24-0203 and GUCC 24-0204.

###### Notes.

Phylogenetic analyses (Fig. 1) revealed that the newly isolated strains (GUCC 24-0202, GUCC 24-0203, and GUCC 24-0204) clustered closely with two strains of *Xenoroussoellatriseptata* (MFLUCC 17-1438 and KNUF-20-NI009). This species was initially reported from dead stems of *Chromolaenaodorata* in Thailand, with its sexual morph documented by Mapook et al. (2020). Subsequently, Ryu et al. (2022) described an asexual morph of this species isolated from soil in Korea. In the current study, sequence data from the ITS, LSU, *ef1-α*, and *rpb2* loci of newly isolated strains were identical to those of *X.triseptata*, confirming their conspecific status. The morphological characteristics of the asexual morph of the new strains were consistent with those previously described by Ryu et al. (2022) based on observations from the PDA medium. Conidiogenous cells were ampulliform to doliiform, producing unicellular conidia. Conidia were globose to ellipsoid with a blunt apex containing one or two guttules. The *conidia* contained one or two guttules, measuring 2.7–5.1 × 1.6–3.3 µm (Ryu et al. 2022). Thus, GUCC 24-0202, GUCC 24-0203, and GUCC 24-0204 were identified as *Xenoroussoellatriseptata*, marking the first recorded occurrence in China.

## ﻿Discussion

Recent extensive surveys across various ecosystems in Guizhou have documented a rich abundance of both saprobic and symbiotic fungi, which play critical ecological roles in nutrient cycling through the decomposition of organic matter (Chuzho et al. 2017; Duan et al. 2022; Luo et al. 2022; Sun et al. 2022, 2023; Wang et al. 2022; Zou et al. 2022; Cai and Zhao 2023; Hyde et al. 2023; Dong et al. 2024; Hu et al. 2024; Wei et al. 2024a, 2024b). Notably, researchers have identified novel and previously unrecorded species, enhancing our understanding of fungal diversity in the province (Dissanayake et al. 2024; Sui et al. 2024; Tian et al. 2024). Our study focused on two families, Didymellaceae and Roussoellaceae, underscoring the significance of Guizhou Province as a hotspot for fungal biodiversity (Wijayawardene et al. 2021a).

Here we introduce *Neoroussoellaguizhouensis* and *Xenodidymellaguizhouensis* based on morphology and multi-gene analyses. *Roussoella* is a speciose genus with 74 species (Yu et al. 2024). During this study we collected three strains of *Roussoellaguizhouensis*, confirming the assumption of Bhunjun et al. (2022) that more species are likely to be discovered in species-rich genera. Furthermore, *Xenoroussoellatriseptata*, previously documented in Korea and Thailand, were reported in China for the first time. In addition, based on the single-gene phylogenetic relationships of ITS, LSU, *rpb2*, and *ef1-α* sequences, we found that in the Roussoellaceae, the single-gene tree of *rpb2* was the most informative gene for determining the phylogenetic position of taxa within these genera. This shows that adding protein-coding genes to the analysis greatly facilitates species-level identification.

## Supplementary Material

XML Treatment for Neoroussoella guizhouensis

XML Treatment for Roussoella guizhouensis

XML Treatment for Xenodidymella guizhouensis

XML Treatment for Xenoroussoella triseptata

## ﻿Additional information

### Author ORCIDs

Chada Norphanphoun https://orcid.org/0000-0002-5756-7206

Kevin D. Hyde https://orcid.org/0000-0002-2191-0762

Fatimah Al-Otibi https://orcid.org/0000-0003-3629-5755

### Data availability

All of the data that support the findings of this study are available in the main text.
